# Management and prognosis of juvenile arthritis on the model of molecular genetic testing of gene p53

**DOI:** 10.1186/1546-0096-12-S1-P162

**Published:** 2014-09-17

**Authors:** Aleksey Kozhevnikov, Nina Pozdeeva, Michael Konev, Valentina Larionova, Gennady Novik

**Affiliations:** 1 Hospital Department of Orthopedics and Rheumatology, The Turner Scientific and Research Institute For Children's Orthopedics, Saint-Petersburg, Russian Federation; 2 Pediatric Department, Saint-Petersburg Pediatric Medical University, Saint-Petersburg, Russian Federation; 3 Genetic Department of Rare Skeletal Disease, The Turner Scientific and Research Institute For Children's Orthopedics, Saint-Petersburg, Russian Federation

## Introduction

Juvenile idiopathic arthritis (JIA) is a multifactor chronic inflammatory joints disease that is characterized by long progressive course leading to development of contractures and loss of joint´s function. Currently, there are only several laboratory and radiological predictors of unfavorable prognosis of JIA. In recent years, scientists studied the molecular basis of the development and maintenance of chronic inflammation in the joint. Reduced sensitivity of cells to apoptosis is one the possible mechanisms contribution to progressive inflammation in synovial membrane. Polymorphisms *Arg72Pro 4 exon*, *ins/del 16bp intron 3* and *G13964C intron 6* gene P53 may change expression gene *P53* and functional activity this protein, main factor of intrinsic apoptosis pathway (P.Dumont et al 2003; A.Sallivan et al, A.Ghosh et al 2004).

## Objectives

Identify predicting factors of course and outcome of JIA in children by based on comprehensive analysis of the clinical and instrumental, laboratory and molecular genetic tests.

## Methods

Clinical, serological, x-ray manifestations, ultrasound and MRI data were analyzed in 126 children with JIA. Three polymorphisms gene *P53* were detected by PCR-RFLP. 60 healthy children without family history of any autoimmune disease were controls.

## Results

We haven't revealed significant differences distribution genotypes of *Arg72Pro ex4*, *ins/del16bp in3* and *G13964C in6* gene *P53* between children with JIA and controls. But girls with oligo- and polyarthritis with genotypes containing three or more minor polymorphic variant *72Pro*, *ins16bp*, *13964C* gene *P53* in any combination were with more severe variant articular lesion. By means of ROC-curve analysis and regression methods were assessed the contribution of molecular genetic, clinical, instrumental and laboratory factors on the disease with a view to determine the prognostic significance of these factors. The presence of erosions of the joints and carrier of genotypes containing allele *72Pro* gene *P53* were found in girls with JIA like highly information signs of prognosis "active" arthritis and the presence of erosions and debut JIA under the age of 3 years old - for boys. Also two signs were obtained for predicting remission in girls with JIA it´s early treatment by DMARS and carrier of homogenous genotype containing allele *Arg72* gene *P53*.

## Conclusion

In order to predict the nature of the disease and to identify children with risk group of the worst course of JIA is possible to use molecular genetic testing of gene *P53* in combination with the study of clinical, instrumental and laboratory data.

## Disclosure of interest

None declared.

